# Supplementary material for: Characterization of an International High-Risk Escherichia coli ST410 Clone Coproducing NDM-5 and OXA-181 in a Food Market in China
Source: Microbiol Spectr. 2023 May 11;11(3):e04727-22. doi: 10.1128/spectrum.04727-22 (PMC10269901; doi:10.1128/spectrum.04727-22)
Supplement: Supplemental file 2 — Supplemental material. Download spectrum.04727-22-s0002.pdf, PDF file, 1.6 MB [file spectrum.04727-22-s0002.pdf]

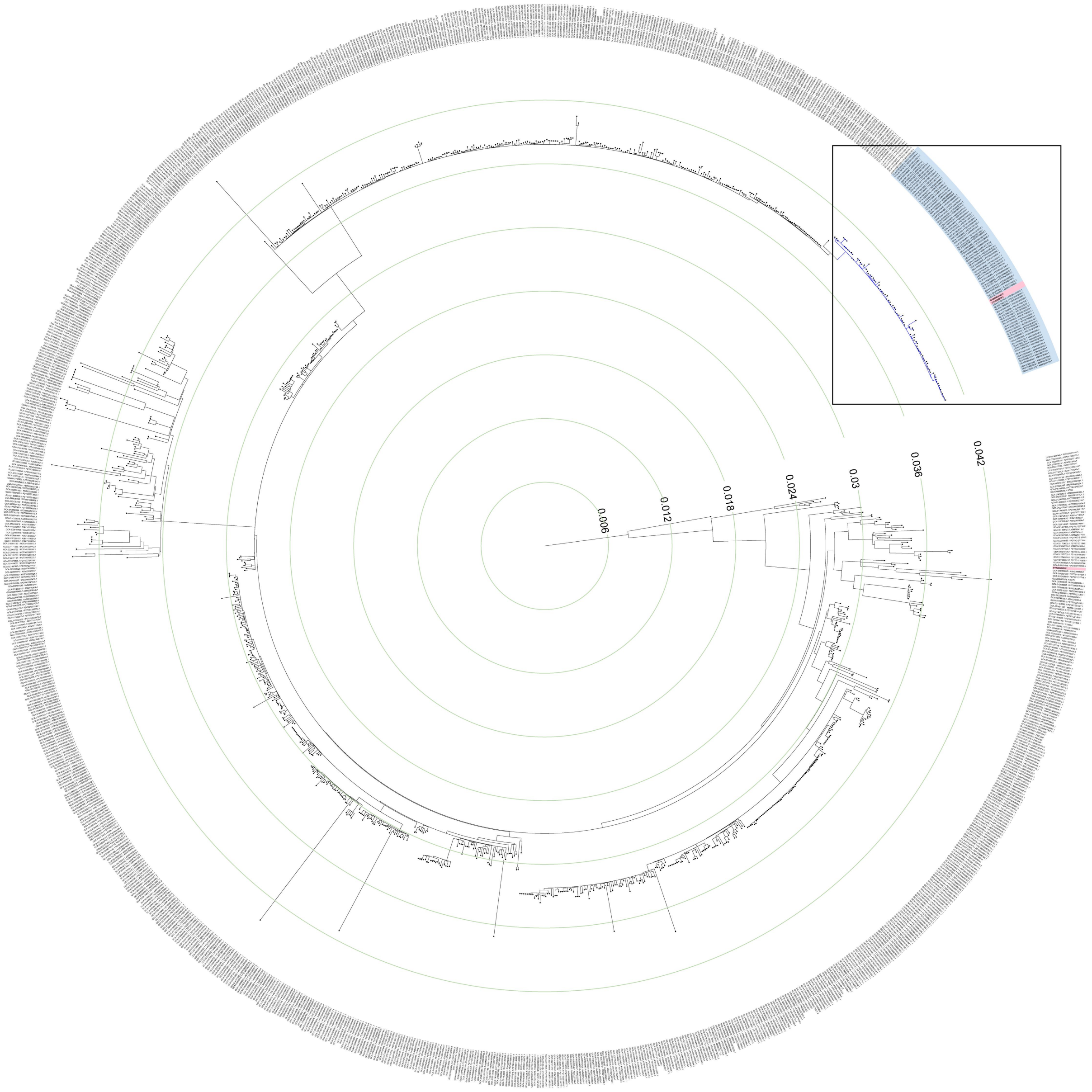

Fig. S1. Phylogenetic tree of *E. coli* ST410 isolates from this study and GenBank (n=1184). Three ST410 isolates characterized in the present study are shaded in pink. GYX208DH4E-2, GYX208DH6-1 and 87 GenBank isolates are clustered into a blue branch.
